# Supplementary material for: Laminin is the ECM niche for trophoblast stem cells
Source: Life Sci Alliance. 2020 Jan 14;3(2):e201900515. doi: 10.26508/lsa.201900515 (PMC6977391; doi:10.26508/lsa.201900515)
Supplement: Supplementary file 1 [file LSA-2019-00515_TableS1.docx]

**Supplemental Table S1. Primers used for quantitative RT-PCR.**

| **Target gene** | **Primer sequences** | **Amplicon size/bp** |
| --- | --- | --- |
| **Integrin α1** | GCCAATGAGACTGTTCCTGAGCTGA | 215 |
|  | TCAAGGCTTCGAGGTCTGCAGTT |  |
| **Integrin α2** | GACTGCAGAACCACTTCCTG | 241 |
|  | CTGCTTTCTCCGTGGGTTTC |  |
| **Integrin α3** | CTGGGGAAGATGCGCATGAGGCATT | 215 |
|  | AAGAAGTACTGGGAGGTCCCTCGTGT |  |
| **Integrin α4** | AGCAGGGCCGACTTAAAGGGA | 312 |
|  | AGCGGGAGTGGGTCGATTGG |  |
| **Integrin α5** | TCAGATCCTCAGCAAGAACC | 212 |
|  | CCTGGTTGATGAGCTCGTAG |  |
| **Integrin α6** | GCCAACCAGAATGGCTCCCAA | 243 |
|  | CACCTGCGAAGGCTTAGCGA |  |
| **Integrin α7x1** | GCACACCTGGATGACGGGCCCTACG | 150 |
|  | CCCTGCCACAAAACTCAGCTCTTCT |  |
| **Integrin α7x2** | ATTGATAGCTCAGACCCTGACCAGC | 162 |
|  | CCCTGCCACAAAACTCAGCTCTTCT |  |
| **Integrin α8** | GCTGCACCCGATGATTATCAACCT | 220 |
|  | GGTGAAATGGGACTGGTGGTTGT |  |
| **Integrin α9** | CTGCATGACAACACTCTCACAC | 194 |
|  | CTGGGACCCATGTTGTAGACC |  |
| **Integrin α10** | TCTTTGTGAGGCTGACTGCCAGTAG | 150 |
|  | AAGGGTGAACCTCATACCGATGCAG |  |
| **Integrin α11** | ATGCCCCACAGAGGAATCACAGCAA | 150 |
|  | TGATCTTCAGAGACCTGTACTTGAG |  |
| **Integrin αV** | CTTGCAGCCCATCCTGAACC | 209 |
|  | GCTCAGCTTCATAGGCACCTT |  |
| **Integrin αIIb** | CTCCAGGCTGGAGCACACCT | 224 |
|  | GGTGATGGACGGGGCGAATG |  |
| **Integrin β1** | GGGTTTCACTTTGCTGGAGATGG | 237 |
|  | TGTGCCCACTGCTGACTTAGG |  |
| **Integrin β3** | TTCCAGCCCACGCTGCAACA | 260 |
|  | GGACGCAGGAGAAGTCATCGCA |  |
| **Integrin β4** | AGCCAGGGAGGCTGGCTTTC | 235 |
|  | GCCTTAACCGTGTATCGGTATGGCT |  |
| **Integrin β5** | GCCAAGATGGCATATCTTACCC | 242 |
|  | TGTTCCATTCCCACTGCATC |  |
| **Integrin β6** | GACTGCTTCAGAAGGATTCTGG | 162 |
|  | TCTTTTGGTGTGGGAAGAGG |  |
| **Integrin β7** | TAGCCCAGGCCCTCACTGCAGCGAA | 150 |
|  | TGATGAGCTGCACCACATTGCTGGA |  |
| **Integrin β8** | GGTGCCATTGCTGGTGAAATAGAAT | 150 |
|  | ATCTGGACAGATGGCAGTGATGTTA |  |
